# Role of Japan's general physicians in healthcare quality improvement and patient safety

**DOI:** 10.1002/jgf2.541

**Published:** 2022-04-01

**Authors:** Takashi Watari, Yasuharu Tokuda

**Affiliations:** ^1^ General Medicine Center Shimane University Shimane Japan; ^2^ Division of Hospital Medicine University of Michigan Health System Ann Arbor Michigan USA; ^3^ Muribushi Okinawa Clinical Training Center Okinawa Japan; ^4^ Tokyo Foundation for Policy Research Tokyo Japan

The importance of diagnostic errors and cognitive biases as part of patient safety issues has been recognized by Japanese general medicine physicians. This has been highlighted in the 2022 ECRI (Emergency Care Research Institute) report, and various activities have been implemented.^1^ However, there has been strong demand for growing roles for generalist physician leaders in hospital quality and safety departments in Japan, but they are able to work only on a part‐time basis because of the routine requirement for clinical work in their area of specialization. Therefore, we believe that the greater focus on medical quality and safety is crucial for Japan's future general physicians. We have delineated our rationale supporting this below.

First, the quality and safety of healthcare are critical to both the “Care” and “Cure” clinical aspects of our delivery. In many cases, healthcare providers have difficulty recognizing that the services they provide may be causing harm to patients. However, the global trend is changing dramatically, with the British Medical Journal announcing in 2016 that medical errors may be the third leading cause of death in the United States.^2^ As illustrated in Figure 1, the level of quality and safety of medical care in Japan is comparable to that in the United States, with more than 100,000 people dying annually due to medical errors. Assuming the number of deaths is lower than the number of accidental events and suicides that fall into the sixth and seventh categories, respectively, there would still be an estimated 30,000 to 40,000 deaths annually. Due to their significant negative impact, medical errors should take precedence over diagnostics and therapeutics when assessing each disease. Fortunately, general medicine physicians in Japan are highly affinitive to social determinants of health, patient engagement, and patient‐centered care. These are essential aspects of quality and safety in healthcare and are highly consistent with the comprehensive viewpoint, including perspective, and their vision.^3^

**FIGURE 1:**
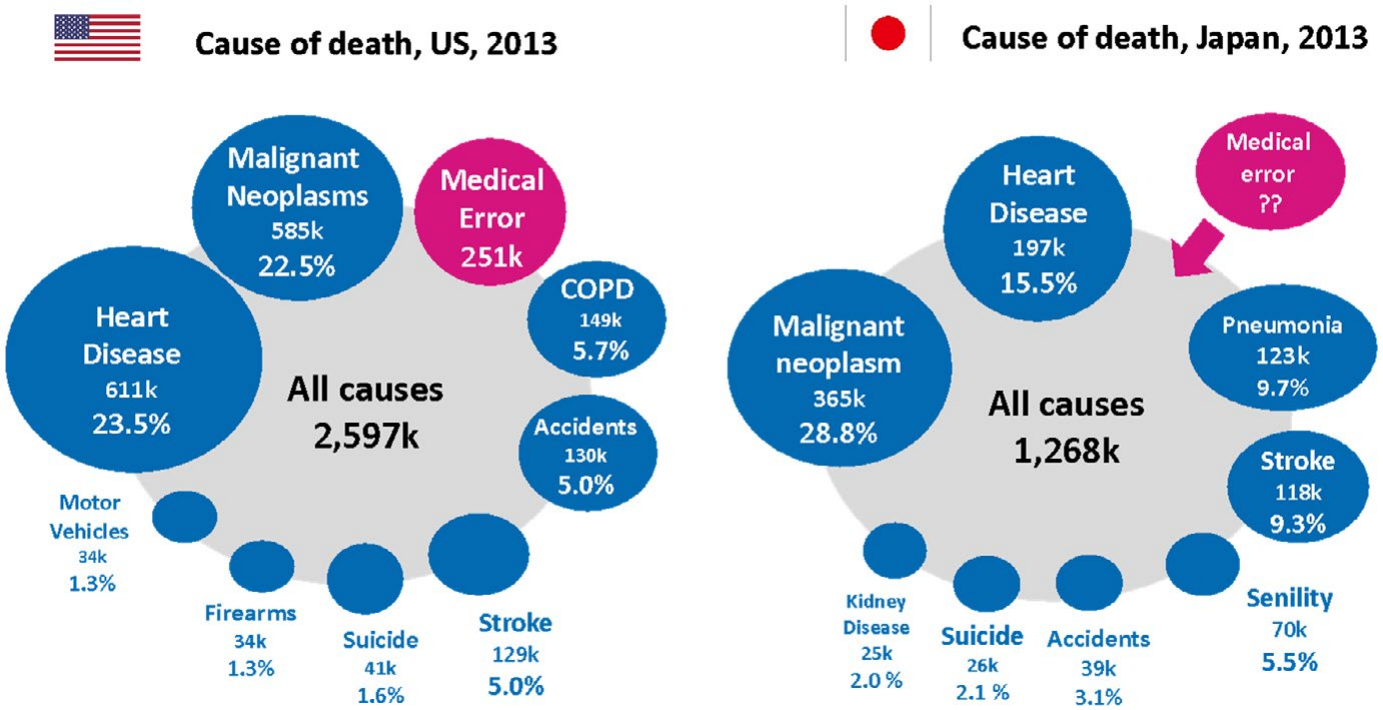
Comparison of medical death causes between United States and Japan in 2013. Prepared by the authors based on the data from Makary MA et al.^2^ and data of “Annual Statistics estimates in 2013” published by the Japanese Ministry of Health, Labour, and Welfare (2014)

Second, the focus on medical quality and safety is highly compatible with the research interests of general practitioners. Many previous studies have shown that medical quality and safety are vital to research areas that spark general physicians' curiosity. This is especially true in facilities where a team provides medical services to patients. The history of Hospital Medicine and General Internal Medicine in the United States shows that general physicians have a strong incentive to improve safety in their practice, cost effectiveness, patient satisfaction, work environment, and educational services at the facility level (micro‐system level).^4^ In addition, the evidence of scientific quality improvement and safety research at the clinical settings (metho‐system level) and regional (metho‐system level), national, and administrative (macro‐system level) levels have had significant impacts. This is one of the main reasons Hospital Medicine has become the largest group of physicians in the United States.^4^ On the downside, in publication data indexed on PubMed by hospitalists that the author (TW) previously published, the quality and safety area of healthcare and the health service area accounts for about 40% of the world's total of the publication by hospitalists.^5^ However, a recent survey conducted by the authors found that the combined research topics of Japanese university hospital general practitioners accounted for less than 2%, and our another study indicated only about 8% of the recent papers published by Japanese general medicine physicians in PubMed between 2015 and 2020 focused on QI and safety area and the health services. This indicates Japanese academic general physicians need to be more active in producing healthcare quality and safety research.^4^, ^5^

Third, general practitioners' skills and knowledge of medical quality and safety will become an important subspecialty.^5^ We believe that the practical knowledge and skills related to QI and safety will be a significant tool for general medicine physicians in future, as much as or more than knowledge of each subspecialty. The “Guidelines for Medical Safety Management” proposed by the Ministry of Health, Labour, and Welfare (MHLW) also urges the establishment of committees and assigning an appropriate leader. However, at present, there is still a dearth of practical experts in this area. In comparison, many of the faculty staff in healthcare quality and safety at Harvard Medical School (MHQS) and the School of Public Health (MPH), the authors learned at, are general physician scientists. Furthermore, the research interests of leading hospitalists in the United States, such as Dr. Sanjay Saint and Dr. Scott Flanders of the University of Michigan, with whom we have a close relationship, are also in healthcare quality and safety.^4^ With effective networking helping us develop our expertise as generalists, it is possible to apply our knowledge in the cross‐disciplinary academic fields where specialists are otherwise not present.^5^

Finally, as management leaders, the authors would like to focus on the usefulness of quality improvement and safety knowledge, and skills for Japanese general physicians.^1^, ^6^ Many general practitioner departments have been established in primary hospitals, university hospitals, or small hospitals in Japan but do not function adequately. The authors are convinced that this expertise in medical quality and safety can be applied to thoroughly analyze the root causes of these failures and implement transformative actions.^1^ Therefore, if various analytical and quality improvement tools in team building, change management, and negotiations can be used, we can accurately identify each complex intertwined cause, predict risks, and develop cost‐effective implementation strategies. These would be great advantages, especially in mid‐level to facility‐wide leadership.

As medicine advances with complexity and sophistication, we have insufficiently kept pace with the performance of our healthcare system when it comes to addressing patient needs, ensuring patient safety, and improving system efficiency and quality. We believe that quality and safety in healthcare will become of more importance than mainstream therapy for diseases in the future. It will be essential for general practitioners, to have an ongoing systematic approach to monitoring, evaluating, and continually improving our care's quality and safety standards. This is where Japanese general physicians can make a difference.

## Funding information

T.W. was supported by grants from the National Academic Research Grant Funds (JSPS KAKENHI: 20H03913). The sponsor of the study had no role in the study design, data collection, analysis, or preparation of the manuscript.

## CONFLICT OF INTEREST

The authors have stated explicitly that there are no conflicts of interest in connection with this article.
